# Impact of Procalcitonin Kinetics on Mortality in Intensive Care Patients with Sepsis

**DOI:** 10.3390/medicina62030487

**Published:** 2026-03-05

**Authors:** Yakup Özgüngör, Burak Emre Gilik, Emre Karagöz, Hicret Yeniay, Mensure Çakırgöz, Özlem Melis Korkmaz Özgüngör, İhsan Birol, Sıla Seven

**Affiliations:** 1 Department of General Intensive Care Unit, İzmir City Hospital, İzmir 35530, Turkey; dr.burakgilik@gmail.com (B.E.G.); dr.emrekaragoz@gmail.com (E.K.); drmensure@gmail.com (M.Ç.); silasevenn@gmail.com (S.S.); 2 Department of General Intensive Care, Balıkesir Atatürk City Hospital, Balıkesir 10185, Turkey; hicret.yeniay@yahoo.com; 3 Department of Occupational and Environmental Diseases, Faculty of Medicine, Dokuz Eylul University, Izmir 35340, Turkey; omlskorkmazoz@gmail.com; 4 Department of Intensive Care, Suat Seren Chest Diseases and Thoracic Surgery Training and Research Hospital, Izmir 35110, Turkey

**Keywords:** sepsis, procalcitonin kinetics, delta procalcitonin, kinetic eGFR, acute kidney injury, mortality prediction

## Abstract

*Background and Objectives*: Procalcitonin (PCT) kinetics are increasingly used as prognostic markers in sepsis, but their interpretation is confounded by dynamic changes in renal function during acute illness. This study evaluated the prognostic value of ΔPCT for 30-day mortality in critically ill patients with either sepsis or septic shock by incorporating serial kinetic eGFR measurements and renal function-adjusted ΔPCT cut-off values based on the mean kinetic eGFR during the first 48–72 h of ICU admission. *Materials and Methods*: This retrospective cohort study included 106 adult ICU patients with either sepsis or septic shock. Serial procalcitonin measurements were used to calculate ΔPCT as a ratio of follow-up to baseline values, while renal function was assessed using mean kinetic eGFR over the first 72 h of ICU admission. *Results*: Thirty-day mortality was 43.4%. ΔPCT was a strong independent predictor of mortality across all models. At 48 h, ΔPCT2 was independently associated with 30-day mortality in the overall cohort (AUC 0.793) and demonstrated independent prognostic significance only in patients with preserved renal function (GFR ≥ 30 mL/min/1.73 m^2^). The optimal ΔPCT2 cut-off corresponded to a 56% reduction in procalcitonin levels. At 72 h, ΔPCT3 emerged as an independent predictor of mortality regardless of renal function. ROC analysis identified an optimal ΔPCT3 cut-off corresponding to 62% procalcitonin reduction in the overall cohort, with renal function-specific thresholds of ~50% for patients with GFR < 30 mL/min/1.73 m^2^ and ~73% for those with preserved renal function. The combination of APACHE II score and ΔPCT3 demonstrated the highest discriminative performance for mortality prediction (AUC 0.948). *Conclusions*: Procalcitonin kinetics provide clinically meaningful prognostic information in sepsis when interpreted alongside dynamic renal function. While 48 h procalcitonin kinetics offer prognostic value primarily in patients with preserved renal function, 72 h ΔPCT provides renal function-independent and superior mortality discrimination. Integrating serial kinetic eGFR measurements enables renal function-adapted ΔPCT threshold determination and may improve risk stratification in critically ill septic patients.

## 1. Introduction

Procalcitonin (PCT) is a 116-amino acid precursor of the hormone calcitonin encoded by the CALC-1 gene. Under physiological conditions, PCT is primarily synthesized by the C cells of the thyroid gland and, to a lesser extent, by neuroendocrine cells and is virtually undetectable in the systemic circulation [1]. However, in response to systemic bacterial infections, transcription of the CALC-1 gene is upregulated in multiple extra-thyroidal tissues, including adipocytes and fibroblasts, under the influence of pro-inflammatory cytokines and bacterial endotoxins such as lipopolysaccharides. This leads to widespread PCT production and release into the bloodstream, resulting in notably elevated plasma concentrations. Elevated PCT levels correlate with the severity of bacterial infection, while declining concentrations are generally associated with clinical improvement and effective antimicrobial therapy [2]. In clinical practice, plasma PCT levels below 0.2 ng/mL are considered to make bacterial sepsis unlikely, whereas values exceeding 0.5 ng/mL warrant further evaluation for possible sepsis [3]. Remarkably elevated PCT levels are predominantly associated with bacterial infections and are less commonly observed in viral or fungal infections.

Sepsis is a life-threatening organ dysfunction caused by a dysregulated host response to infection and represents a major cause of morbidity and mortality worldwide. If not recognized and treated promptly, sepsis may progress to septic shock, multiple organ dysfunction syndrome, and subsequently death. Although a wide spectrum of pathogens can trigger sepsis, bacterial infections remain the most common etiology compared to viral or fungal causes [4].

In this context, PCT has emerged as a valuable biomarker for the assessment and monitoring of critically ill patients with sepsis in the intensive care unit (ICU). Plasma PCT concentrations typically start off rising within the first 2 to 6 h following exposure to an inflammatory stimulus, peak at approximately 12 to 24 h, and exhibit a biological half-life of about 24 h, although reported ranges vary between 22 and 35 h [5]. Eminently, renal function plays a critical role in measuring the circulating PCT levels. Given its relatively small molecular weight (approximately 14.5 kDa), PCT is, at least, partially cleared by the kidneys, and impaired renal function may lead to delayed clearance and persistently elevated plasma levels [6]. Both acute kidney injury (AKI) and chronic kidney disease (CKD) have been shown to affect baseline PCT concentrations and alter its kinetic profile, thereby complicating interpretation based on single measurements [7].

In critically ill patients, particularly those with sepsis or septic shock, renal function often changes swiftly during the early phase of hospitalization, rendering a single baseline estimated glomerular filtration rate (eGFR) insufficient to accurately reflect true renal clearance capacity. In order to address this limitation, the concept of kinetic estimated glomerular filtration rate (kinetic eGFR) has been proposed, which incorporates the rate of change in serum creatinine over time and provides a dynamic assessment of renal function during non-steady-state conditions [8]. In the context of sepsis, where both inflammatory burden and renal clearance directly influence circulating biomarker levels, the use of kinetic eGFR may allow a more physiologically appropriate interpretation of procalcitonin kinetics. Accordingly, integrating serial kinetic eGFR measurements into the evaluation of procalcitonin dynamics may reduce misclassification related to fluctuating renal function and improve prognostic stratification in septic patients.

Therefore, this study aimed to investigate procalcitonin kinetics in critically ill patients with sepsis or septic shock, and to evaluate the association between ΔPCT and mortality by incorporating serial kinetic eGFR measurements to account for dynamic changes in renal function.

## 2. Materials and Methods

This retrospective observational study was conducted in the 120-bed General Intensive Care Unit (ICU) of İzmir City Hospital, a tertiary-care center admitting both medical and surgical critically ill patients. The study population comprised of all consecutive patients hospitalized between 1 January and 31 March 2025, with a diagnosis of either sepsis or septic shock in accordance with contemporary Sepsis-3 definitions. Patients aged 18 years old or above with a minimum ICU stay of 72-h, availability of numerous serial biochemical analyses at 24-h intervals including procalcitonin (PCT), and documented APACHE II and SOFA scores on ICU admission were eligible for inclusion. Patients with a recent history of cardiopulmonary arrest prior to admission, major trauma, recent surgery, or malignancy-associated PCT elevation were excluded.

The adequacy of empiric antibiotic therapy was evaluated based on microbiological culture results. Patients diagnosed with sepsis but without microbial growth in culture were classified as “unknown.” Patients who received empiric antibiotic therapy that was active against the isolated pathogen according to culture susceptibility results were classified as “adequate,” whereas those in whom the empirically selected antibiotic was resistant to the cultured pathogen were classified as “inadequate.”

The investigation was conducted in two sequential phases. In the initial phase, the association between serial procalcitonin changes at 24, 48, and 72 h relative to baseline values on ICU admission and 30-day mortality was evaluated, and the incremental prognostic value of ΔPCT beyond established severity scores (APACHE II and SOFA) was assessed. ΔPCT was initially calculated as the logarithmic ratio of follow-up PCT values to baseline values to standardize the distribution and minimize the influence of extreme values; subsequently, percentage changes in PCT were derived to facilitate clinically intuitive interpretation in routine practice. ΔPCT1, ΔPCT2, and ΔPCT3 were defined as the logarithmic ratios of serum procalcitonin concentrations measured at 24, 48, and 72 h, respectively, to baseline values obtained on ICU admission (ΔPCT = log_10_[PCT_follow-up/PCT_baseline]). Daily GFR values were calculated by the hospital laboratory using the 2021 CKD-EPI equation, and these data were drawn from the electronic medical record. Subsequently, to capture dynamic changes in renal function, the kinetic estimated glomerular filtration rate (kinetic GFR) was calculated using the validated kinetic GFR formula based on serial serum creatinine measurements obtained during the first 72 h of ICU admission. Kinetic GFR was calculated according to the validated kinetic GFR formula [8]:

$$\mathrm{kGFR}=\frac{\left({\mathrm{SCr}}_{\mathrm{baseline}}\times {\mathrm{SCr}}_{0}\right)}{\left(\frac{{\mathrm{SCr}}_{72h}\;+\;{\mathrm{SCr}}_{0}}{2}\right)}\times (1-\frac{24\times ({\mathrm{SCr}}_{72h}-{\mathrm{SCr}}_{0})}{72\times 1.5})$$

where **SCr_0_** represents the serum creatinine concentration at ICU admission, **SCr_72h_** the creatinine concentration at 72 h, and 1.5 mg/dL/day denotes the maximal theoretical rate of creatinine increase. This formula estimates real-time glomerular filtration by incorporating both baseline creatinine concentration and its rate of change over time, thereby providing a dynamic assessment of renal function that is more sensitive to acute fluctuations than conventional static creatinine-based equations.

Patients were subsequently stratified into renal function groups according to kinetic GFR values, and renal function-specific ΔPCT thresholds for discriminating survivors from non-survivors were determined using receiver operating characteristic (ROC) curve analysis with the Youden index.

All statistical analyses were performed using SPSS version 26.0 for Windows (IBM Corp., Armonk, NY, USA). Continuous variables were summarized as mean ± standard deviation, and categorical variables as counts and percentages. Between-group comparisons were conducted using Student’s *t*-test or Mann–Whitney U test for continuous variables and the χ^2^ test or Fisher’s exact test for categorical variables, as appropriate. Variables associated with 30-day mortality at a significance level of *p* < 0.05 in univariate analyses were entered into a binary logistic regression model. Predictive performance was evaluated using ROC curve analysis.

The study protocol was approved by the İzmir City Hospital Non-Interventional Clinical Research Ethics Committee (approval number: 2025/1847). Due to the retrospective design of the study, the requirement for informed consent was waived.

Generative artificial intelligence tools were used solely for language editing and improvement of manuscript clarity. No AI tools were used for study design, data collection, data analysis, interpretation of results, or figure generation. All scientific content was reviewed and approved by the authors.

## 3. Results

A total of 106 patients were included in the study. Of these, 64.2% were male; 71.7% were admitted with sepsis and 28.3% with septic shock. The prevalence of diabetes mellitus was 49.1%, hypertension 61.3%, chronic kidney disease 38.7%, and chronic obstructive pulmonary disease 31.1%. Infections were hospital-acquired in 60.4% of cases, and blood cultures were positive in 39.6%, with Gram-negative bacteria accounting for 21.7% of isolates. Acute kidney injury occurred in 72.6% of patients, and 39.6% were classified as AKI stage 3. The 30-day mortality rate was 43.4%. Baseline demographic and clinical characteristics of the study cohort are summarized in Table 1.

When continuous variables were compared according to mortality status, APACHE II scores were higher in non-survivors than in survivors (37.54 ± 11.58 vs. 21.15 ± 7.22; *p* < 0.001). SOFA scores were also higher in the non-survivor group (8.71 ± 3.50 vs. 6.60 ± 2.94; *p* = 0.001). Baseline procalcitonin values differed significantly between groups (*p* = 0.027). Baseline creatinine (*p* = 0.086) and baseline estimated GFR (*p* = 0.086) did not differ significantly; however, mean kinetic GFR was lower in non-survivors (38.30 ± 27.04 vs. 56.71 ± 32.43; *p* = 0.007). Survivors exhibited a greater proportional drop in procalcitonin levels over 72 h (*p* < 0.001). Descriptive statistics and group comparisons for continuous variables are presented in Table 2.

Multivariable logistic regression analyses were performed to evaluate the prognostic performance of procalcitonin kinetics at 24, 48, and 72 h. At 24 h, adjustment for APACHE II demonstrated that APACHE II remained an independent predictor of 30-day mortality, whereas ΔPCT1 did not possess statistical significance despite a large effect size (OR = 15.58, *p* = 0.067). In contrast, when adjusted for SOFA score, both SOFA and ΔPCT1 were independently associated with mortality (ΔPCT1: OR = 32.43, *p* = 0.003; SOFA: OR = 1.24, *p* = 0.003).

At 48 h, models incorporating ΔPCT2 demonstrated statistical significance. The combination of APACHE II and ΔPCT2 displayed a strong association with mortality (*p* < 0.001; Nagelkerke R^2^ = 0.628; overall accuracy = 82.1%), with both APACHE II (OR = 1.179, *p* < 0.001) and ΔPCT2 (OR = 20.48, *p* = 0.003) remaining independently associated with 30-day mortality. Calibration was adequate (Hosmer–Lemeshow *p* = 0.797), and bootstrap validation confirmed the stability of both predictors. Similarly, the model including SOFA score and ΔPCT2 was statistically significant (*p* < 0.001; Nagelkerke R^2^ = 0.378; accuracy = 78.3%), with independent associations observed for ΔPCT2 (OR = 22.72, *p* < 0.001) and SOFA score (OR = 1.19, *p* = 0.020), both of which were confirmed by bootstrap analysis.

At 72 h, ΔPCT3 showed associations with 30-day mortality across all models. When combined with APACHE II, the model demonstrated strong explanatory power (*p* < 0.001; Nagelkerke R^2^ = 0.743; accuracy = 84.0%), with both ΔPCT3 (OR = 72.77, *p* < 0.001) and APACHE II (OR = 1.21, *p* < 0.001) remaining independently associated with mortality; these findings were confirmed by bootstrap validation. In the model adjusted for SOFA score, ΔPCT3 remained statistically significant (OR = 45.21, *p* < 0.001), whereas SOFA score did not possess significance after adjustment (*p* = 0.099), a result also reflected in bootstrap analysis. Multivariable regression results are summarized in Table 3.

**Figure 1:**
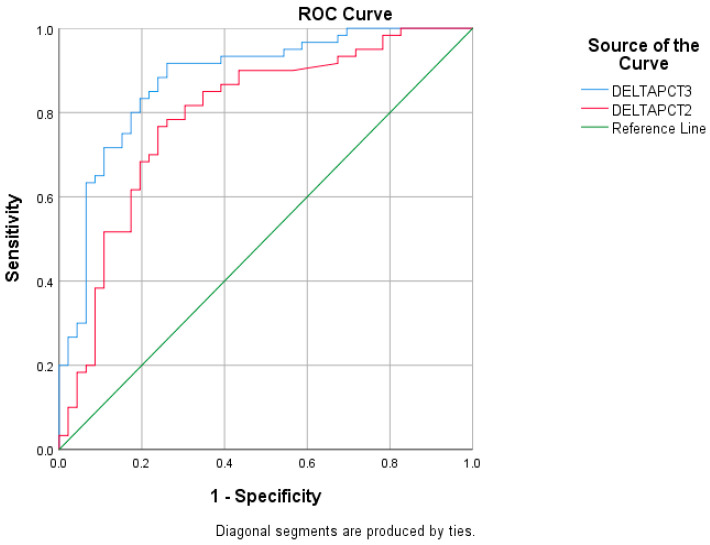
ROC curve for 48 and 72 h procalcitonin decrease.

Receiver operating characteristic (ROC) analysis was performed to determine the optimal cut-off values for procalcitonin kinetics at 48 and 72 h using the Youden index. For ΔPCT2 (48 h), the optimal cut-off value was −0.36, corresponding to an approximate 56% reduction in serum procalcitonin levels relative to baseline (10^−0.36^ ≈ 0.44), with a sensitivity of 63.3% and a specificity of 80.4%. For ΔPCT3 (72 h), the optimal cut-off was −0.42, corresponding to an approximate 62% reduction in procalcitonin levels from baseline (10^−0.42^ ≈ 0.38), providing a sensitivity of 80.0% and a specificity of 80.4%. ROC analysis results are shown in Table 4 and Figure 1.

In multivariable logistic regression analyses adjusted for renal function, procalcitonin kinetics at 48 h demonstrated a differential prognostic impact according to renal function. While the APACHE score remained an independent predictor of 30-day mortality in both renal subgroups (GFR < 30: *p* = 0.010; GFR ≥ 30: *p* < 0.001), ΔPCT2 was independently associated with mortality only among patients with preserved renal function (GFR ≥ 30 mL/min/1.73 m^2^; *p* = 0.034), an association that remained robust after bootstrap resampling (*p* = 0.017). In contrast, in patients with impaired renal function (GFR < 30 mL/min/1.73 m^2^), ΔPCT2 failed to retain independent prognostic significance (*p* = 0.114; bootstrap *p* = 0.113), indicating limited clinical utility of early procalcitonin kinetics in this subgroup.

In contrast to 48 h procalcitonin kinetics, ΔPCT3 at 72 h remained an independent predictor of 30-day mortality in both renal subgroups after adjustment for disease severity. In multivariable models incorporating the APACHE score, ΔPCT3 demonstrated robust and consistent prognostic significance in patients with lower renal function (GFR < 30 mL/min/1.73 m^2^; *p* = 0.018; bootstrap *p* = 0.003) as well as in those with preserved renal function (GFR ≥ 30 mL/min/1.73 m^2^; *p* = 0.003; bootstrap *p* = 0.001). The inclusion of ΔPCT3 substantially improved model performance and discrimination across both groups.

Receiver operating characteristic (ROC) analysis stratified by renal function was performed to determine optimal cut-off values for procalcitonin kinetics at 48 and 72 h using the Youden index. In patients with impaired renal function (GFR < 30 mL/min/1.73 m^2^), ROC discrimination for ΔPCT2 at 48 h was insufficient (AUC = 0.724, *p* = 0.018), and therefore this parameter was not considered clinically meaningful and was excluded from further cut-off interpretation.

Conversely, among patients with preserved renal function (GFR ≥ 30 mL/min/1.73 m^2^), the optimal Youden-based cut-off for ΔPCT2 at 48 h was −0.35, corresponding to an approximate 55% reduction in procalcitonin levels (10^−0.35^ ≈ 0.45), with a sensitivity of 77.3% and a specificity of 65.2%, whereas the optimal ΔPCT3 cut-off at 72 h was −0.57, corresponding to an approximate 73% reduction from baseline (10^−0.57^ ≈ 0.27), providing a sensitivity of 84.1% and a specificity of 78.3% (Table 5).

## 4. Discussion

As expected, in the first phase of the study, a decline in procalcitonin levels following treatment was predictive of 30-day mortality. A recent similar study also suggested that drops in procalcitonin can be utilized to guide therapy and to determine antibiotic duration through daily procalcitonin monitoring in conjunction with standard antimicrobial regimens [9]. In this regard, the observed association between procalcitonin reduction and mortality is consistent with and meaningful within the existing literature.

Patients admitted to the intensive care unit with either sepsis or septic shock frequently present with acute kidney injury, chronic kidney disease, or acute-on-chronic renal dysfunction. In the present study, 72.6% of patients exhibited some degree of renal impairment. Conventional GFR estimation methods, including the widely used CKD-EPI 2021 equation, are based on single serum creatinine measurements and assume a steady-state creatinine level [10]. However, in conditions such as sepsis, renal function may change promptly over short time intervals. For this reason, approaches incorporating both baseline and serial creatinine measurements have been recommended instead of relying on a single cross-sectional GFR estimate [8]. Accordingly, our study demonstrates that the rate of procalcitonin decline varies in alliance with dynamic changes in renal function during treatment. The threshold of GFR < 30 mL/min/1.73 m^2^ was adopted based on prior studies investigating renal function and procalcitonin clearance [11,12]. From this perspective, our study conveys two clinically important findings. First, in patients with GFR < 30 mL/min/1.73 m^2^, an adequate procalcitonin response tends to occur only after 48 h. Second, even at 72 h, the decline in procalcitonin remains more gradual in patients with impaired renal function. Consequently, procalcitonin-based antibiotic modifications during the first 48 h may be misleading in patients with GFR < 30 mL/min/1.73 m^2^.

The main strengths of this study include the utilization of procalcitonin kinetics rather than static measurements, the integration of ΔPCT with established severity scores, and the novel application of serial kinetic eGFR to contextualize biomarker interpretation under dynamically changing renal function.

Several limitations ought to be acknowledged. First, the single-center, retrospective design limits causal inference and may introduce center-specific bias related to local microbiological epidemiology, antimicrobial resistance patterns, and institutional treatment strategies, thereby restricting generalizability to other ICU settings. The high prevalence of acute kidney injury in the present cohort (72.6%) reflects the severity profile of this tertiary-care population and may not represent less severely ill or mixed ICU populations. Procalcitonin measurement timing was determined by routine clinical practice rather than a fully standardized sampling protocol, which may have introduced variability in ΔPCT calculation despite the use of predefined time windows. Furthermore, subgroup analyses stratified by renal function—particularly within intermediate kinetic eGFR ranges—were limited by sample size and event distribution. This constraint may have contributed to coefficient instability and wider confidence intervals in regression models; therefore, the proposed renal function-adjusted ΔPCT thresholds should be interpreted as exploratory and hypothesis-generating rather than definitive clinical cut-off values. Prominently, although 72 h ΔPCT kinetics demonstrated strong discriminative performance in this cohort, their role should be considered prognostic rather than decision-guiding at this stage, as early mortality assessment remains crucial in sepsis management. While internal validation using bootstrapping techniques was performed to assess model stability and reduce optimism bias, external validation was not feasible. Accordingly, prospective multicenter studies are required to confirm the stability, reproducibility, and clinical applicability of the proposed renal function-adjusted ΔPCT approach before integration into routine bedside decision-making. Finally, not all potential confounders—such as timing of source control, antimicrobial appropriateness, and infection focus—could be fully accounted for, leaving the possibility of residual confounding.

## 5. Conclusions

ΔPCT provides significant prognostic information for 30-day mortality in critically ill patients with either sepsis or septic shock, and its predictive performance is enhanced when interpreted in conjunction with clinical severity scores and renal function assessed by kinetic eGFR.

## Figures and Tables

**Table 1:** Distribution of categorical variables in the study cohort (n = 106).

Variable	Categories	n (%)
Sex	Female	38 (35.8)
	Male	68 (64.2)
Sepsis category	Sepsis	76 (71.7)
	Septic shock	30 (28.3)
Diabetes mellitus	Present	52 (49.1)
	Absent	54 (50.9)
Hypertension	Present	65 (61.3)
	Absent	41 (38.7)
COPD	Present	33 (31.1)
	Absent	73 (68.9)
Chronic kidney disease	Present	41 (38.7)
	Absent	65 (61.3)
Coronary artery disease	Present	34 (32.1)
	Absent	72 (67.9)
Source of infection	Community-acquired	42 (39.6)
	Hospital-acquired	64 (60.4)
Blood culture result	No growth	57 (53.8)
	Growth present	42 (39.6)
	Culture not obtained	7 (6.6)
Pathogen type	No pathogen detected	62 (58.5)
	Gram-negative	23 (21.7)
	Gram-positive	12 (11.3)
	Fungal	8 (7.5)
	Viral	1 (0.9)
Empiric antibiotic adequacy	Adequate	34 (32.1)
	Inadequate	9 (8.5)
	Unknown	62 (58.5)
RRT modality	None	75 (70.8)
	IHD	13 (12.3)
	CRRT	7 (6.6)
	Chronic HD patient	11 (10.4)
AKI stage	No AKI	29 (27.4)
	Stage 1	13 (12.3)
	Stage 2	10 (9.4)
	Stage 3	42 (39.6)
	Chronic HD	12 (11.3)
AKI outcome	No AKI	31 (29.2)
	Complete recovery	27 (25.5)
	Partial recovery	7 (6.6)
	ESRD	9 (8.5)
	AKI-related death	32 (30.2)
30-day mortality	Survived	60 (56.6)
	Died	46 (43.4)

Data are presented as numbers (%). AKI: acute kidney injury; CKD: chronic kidney disease; COPD: chronic obstructive pulmonary disease; RRT: Renal Replacement Therapy; HD: Hemodialysis.

**Table 2:** Continuous variables compared between survivors and non-survivors.

Variable	Survivors Mean ± SD	Non-Survivors Mean ± SD	*p*-Value
Age (years)	68.92 ± 16.82	73.17 ± 12.87	0.431
APACHE II	21.15 ± 7.22	37.54 ± 11.58	<0.001
SOFA	6.60 ± 2.94	8.71 ± 3.50	0.001
Procalcitonin (baseline)	24.00 ± 32.24	18.56 ± 32.83	0.027
Creatinine (baseline)	2.18 ± 1.86	2.61 ± 1.89	0.086
Estimated GFR (baseline)	52.33 ± 33.79	37.33 ± 29.66	0.086
MeanGFR (kinetic)	56.71 ± 32.43	38.30 ± 27.04	0.007
DeltaPCT1 (log10)	−0.1668 ± 0.1948	−0.0103 ± 0.2636	0.002
DeltaPCT2 (log10)	−0.4157 ± 0.2868	0.0080 ± 0.5158	<0.001
DeltaPCT3 (log10)	–0.7684 ± 0.4349	0.0943 ± 0.7413	<0.001
Albumin (baseline)	30.38 ± 6.21	28.36 ± 5.54	0.092
Days until death	2.37 ± 10.33	9.41 ± 6.58	0.608

Values are expressed as mean ± standard deviation. Group comparisons were performed using Student’s *t*-test or the Mann–Whitney U test, as appropriate based on data distribution. ΔPCT values were calculated as the logarithmic ratios of procalcitonin concentrations measured at 24, 48, and 72 h to baseline values obtained on ICU admission (ΔPCT = log_10_[PCT_follow-up/PCT_baseline]).

**Table 3:** Multivariable logistic regression models predicting 30-day mortality.

Model	*p*-Value	OR (Exp B)	95% CI	Accuracy (%)
Model 1 (APACHE II + ΔPCT2)	<0.001	20.48	2.92–143.70	82.1
Model 2 (SOFA + ΔPCT2)	<0.001	22.72	4.18–123.59	78.3
Model 1 (APACHE II + ΔPCT3)	<0.001	1.202	1.10–1.32	84
Model 2 (SOFA + ΔPCT3)	<0.001	42.718	9.04–201.89	84

OR, odds ratio; CI, confidence interval. ΔPCT2 and ΔPCT3 were calculated as the logarithmic ratios of serum procalcitonin concentrations at 48 and 72 h, respectively, to baseline values obtained on ICU admission (ΔPCT2 = log_10_[PCT_48_h/PCT_0_h]; ΔPCT3 = log_10_[PCT_72_h/PCT_0_h]). Multivariable logistic regression models were constructed using APACHE II or SOFA scores in combination with ΔPCT, as well as ΔPCT alone. Model accuracy represents the overall correct classification rate.

**Table 4:** Area under the ROC curve for mortality prediction.

Predictor	AUC	Std Error	*p*-Value	95% CI
APACHE II + ΔPCT2	0.904	0.029	<0.001	0.846–0.961
SOFA + ΔPCT2	0.814	0.044	<0.001	0.728–0.900
ΔPCT2	0.793	0.046	<0.001	0.703–0.883
APACHE II	0.877	0.037	<0.001	0.804–0.950
SOFA	0.676	0.054	0.002	0.569–0.782
APACHE II + ΔPCT3	0.948	0.018	<0.001	0.912–0.984
SOFA + ΔPCT3	0.885	0.032	<0.001	0.821–0.948
ΔPCT3	0.878	0.035	<0.001	0.810–0.946

AUC, area under the curve; CI, confidence interval. ROC *p*-values test the null hypothesis that the true AUC equals 0.5. ΔPCT2 and ΔPCT3 were calculated as log_10_ ratios of serum procalcitonin levels at 48 and 72 h, respectively, to baseline values obtained on ICU admission.

**Table 5:** Optimal ΔPCT thresholds expressed as logarithmic ratios and absolute percentage decrease in procalcitonin.

Group	Time Point	ΔPCT Cut-Off (log10)	Ratio (10^ΔPCT^)	Required PCT Decline (%)
All patients	48 h (ΔPCT2)	−0.36	0.44	56.0% decrease
All patients	72 h (ΔPCT3)	−0.42	0.38	62.0% decrease
GFR < 30 mL/min/1.73 m^2^	72 h (ΔPCT3)	−0.30	0.50	50.0% decrease
GFR ≥ 30 mL/min/1.73 m^2^	48 h (ΔPCT2)	−0.35	0.45	55.0% decrease
GFR ≥ 30 mL/min/1.73 m^2^	72 h (ΔPCT3)	−0.57	0.27	73.0% decrease

## Data Availability

The data supporting the findings of this study are available from the corresponding author upon reasonable request. Due to ethical and institutional restrictions related to patient privacy and data protection regulations, the data are not publicly available.

## Abbreviations

The following abbreviations are used in this manuscript:

AKI	Acute kidney injury
APACHE II	Acute Physiology and Chronic Health Evaluation II
AUC	Area under the curve
CI	Confidence interval
CKD	Chronic kidney disease
COPD	Chronic obstructive pulmonary disease
CRRT	Continuous renal replacement therapy
ΔPCT	Delta procalcitonin ((log_10_[PCT_follow-up/PCT_baseline])
eGFR	Estimated glomerular filtration rate
ESRD	End-stage renal disease
GFR	Glomerular filtration rate
ICU	Intensive care unit
IHD	Intermittent hemodialysis
kGFR	Kinetic estimated glomerular filtration rate
OR	Odds ratio
PCT	Procalcitonin
ROC	Receiver operating characteristic
SD	Standard deviation
SOFA	Sequential Organ Failure Assessment
